# Healthcare professionals’ perspectives toward innovation in palliative care across European settings: an exploratory mixed-methods study

**DOI:** 10.3389/fpubh.2026.1934362

**Published:** 2026-09-23

**Authors:** Sara Cruz, Mariana Silva-Ferreira, Maria Cândida Silva, Michael Sapateiro Luís, Sara Monteiro-Reis, Rui Henrique, Carmen Jerónimo, Ambre Laplaud, Matthieu Frasca, Elixabete Elordi Astegia, Rosana Angles Barbastro, Fátima González Palau, Marta García Rodriguez, Raquel Losada Durán

**Affiliations:** 1 Doctoral Program in Nursing Sciences, ICBAS – School of Medicine and Biomedical Sciences – University of Porto, Porto, Portugal; 2 Oncology Nursing Research Unit, IPO Porto Research Center (CI-IPO Porto), Portuguese Oncology Institute of Porto (IPO Porto), Porto, Portugal; 3 RISE—Associate Laboratory (Health Research Network), Porto, Portugal; 4 Porto Comprehensive Cancer Center Raquel Seruca (Porto.CCC), Porto, Portugal; 5 Department of Surgical Oncology, Portuguese Oncology Institute of Porto (IPO Porto), Porto, Portugal; 6 Cancer Biology & Epigenetics Group, IPO Porto Research Center (CI-IPO Porto), Portuguese Oncology Institute of Porto (IPO Porto), Porto, Portugal; 7 Clinical Oncology Group, IPO Porto Research Center (CI-IPO Porto), Portuguese Oncology Institute of Porto (IPO Porto), Porto, Portugal; 8 Department of Palliative Care, Portuguese Oncology Institute of Porto (IPO Porto), Porto, Portugal; 9 ULSNE—Unidade Local de Saúde Nordeste, Bragança, Portugal; 10 Department of Pathology and Molecular Immunology, ICBAS—School of Medicine and Biomedical Sciences—University of Porto, Porto, Portugal; 11 Department of Pathology, Portuguese Oncology Institute of Porto (IPO Porto), Porto, Portugal; 12 Gérontopôle Nouvelle-Aquitaine, Limoges, France; 13 CHU de Bordeaux, Service d'accompagnement et de Médecine Palliative, Bordeaux, France; 14 Inserm U1219, Equipe EPIdémiologie du Cancer et des Expositions Environnementales EPICENE, Université de Bordeaux, Bordeaux, France; 15 Diputación Foral de Bizkaia, Regional Government of Bizkaia, Bilbao, Bizkaia, Spain; 16 Servicio Aragonés de Salud, Hospital de Barbastro, Barbastro, Spain; 17 Instituto de Neurociencias y Bienestar, Universidad Siglo 21, Córdoba, Argentina; 18 Fundación INTRAS, Valladolid, Spain

**Keywords:** digital health, healthcare professionals, implementation, innovation, palliative care

## Abstract

Innovation in palliative care often relies on technological advancements, yet its implementation depends on complex organizational and systemic conditions. An exploratory cross-sectional mixed-methods survey study was conducted to investigate healthcare professionals’ perspectives on innovation across multiple European contexts. Survey data assessed perceived implementation, openness to innovation, management support, and familiarity versus use of digital tools, complemented by qualitative open-ended responses. Findings suggest that while participants demonstrated high conceptual openness to innovation, defining it as a multidimensional process encompassing communication, continuity of care, and service reorganization, a marked awareness-implementation gap was identified. Even though familiarity with digital technologies is widespread, routine use remains limited. Barriers were primarily structural, including funding instability, workforce pressures, fragmented infrastructure, and insufficient institutional alignment, rather than ideological resistance. This study underscores that sustainable digital transformation in palliative care requires strengthening system readiness, integrating infrastructure investment, leadership engagement, and human-centered implementation strategies to ensure innovation reinforces relational models of care.

## 1 Introduction

Healthcare systems worldwide are undergoing significant changes driven by population ageing, rising rates of multimorbidity, workforce shortages, and escalating care complexity. In Europe, these demographic and epidemiological shifts have led to an intensified demand for integrated, person-centered innovative models of care that can address serious and life-limiting illnesses (1). Palliative care (PC) is now widely recognized as an essential health service that improves quality of life, reduces unnecessary hospitalizations, and supports dignity at the end of life (1). However, access to PC remains uneven, often under-resourced and structurally fragmented across different levels of care.

Within this context, digital health has emerged as a key strategy to address these challenges by creating new ways to improve continuity of care, symptom management, and support for patients and families across settings. In PC, technological innovations, including telehealth programs, digital symptom-monitoring platforms, interoperable electronic health records, and decision-support tools, have shown potential for extending care beyond institutional boundaries and supporting home-based care (2). These technologies are now seen as critical components of the ongoing transformation of contemporary health systems.

Despite these opportunities, the integration of digital health technologies into PC presents unique challenges. PC is intrinsically relational, relying on communication, ethical deliberation, shared decision-making, and emotional support. As such, innovation in this field must align with professional values and care philosophies while preserving human presence, compassion, and dignity (3). Understanding how digital tools are perceived, adopted, and integrated into everyday practice is therefore essential.

From a systems perspective, healthcare is increasingly conceptualized as a complex adaptive system, in which successful innovation depends on dynamic interactions between technologies, professionals, organizations, and sociocultural contexts (4). Implementation science highlights that the adoption and normalization of health innovations are shaped by multilevel determinants, including organizational culture, leadership, training opportunities, resource availability, and perceived usefulness (5). Without alignment across these areas, digital health technologies often remain limited to pilot initiatives or to achieve widespread adoption. In PC, where ethical sensitivity and relational continuity are central, tensions between technological transformation and humanistic values may intensify implementation challenges.

Existing literature on PC has predominantly focused on developing and evaluating the feasibility and effectiveness of specific digital interventions, such as telehealth programs, remote monitoring tools, digital advanced-care planning tools, and clinical decision-support platforms, often reporting positive outcomes (6). While these studies are important for demonstrating clinical value, less attention has been devoted to understanding how frontline professionals conceptualize innovation, perceive digital health technologies, and experience the contextual factors that facilitate or hinder their implementation in routine practice (7). Professional attitudes, openness to change, and confidence in using digital tools are widely recognized as critical yet underexplored determinants of implementation success.

There is also limited evidence from cross-national studies on innovation in PC. Differences in health system organization, digital infrastructure maturity, funding mechanisms, and regulatory frameworks across countries can significantly influence innovation pathways and implementation readiness (8). A look at these issues from a territorial perspective may identify contextual enablers and structural bottlenecks that might remain obscured in single-site or mono-national studies. Exploring these dynamics within the European landscape offers an opportunity to identify shared challenges and context-specific drivers of digital health integration.

Considering this background, we aimed to explore healthcare professionals’ attitudes toward innovation in PC, including both technological and non-technological forms of change, across different European settings. Accordingly, a cross-sectional mixed-methods survey was conducted within the HENKO-NET network. Specifically, we examined perceived levels of innovation, barriers and facilitators to implementation, knowledge and use of digital health technologies, and future-oriented perspectives. Ultimately, and by integrating quantitative and qualitative data, we sought to provide a comprehensive and practice-informed understanding of the conditions required for human-centered, sustainable digital health innovation in PC.

## 2 Materials and methods

### 2.1 Study design

An exploratory cross-sectional mixed-methods study was conducted to investigate the perceptions of health professionals working in PC regarding innovation, barriers and facilitators. A structured questionnaire was developed specifically aimed at this target group.

### 2.2 Questionnaire development

The process of constructing the questionnaire for this exploratory study involved several steps to ensure the relevance, clarity, and validity of the content. Initially, a comprehensive literature review was conducted to identify the key topics and relevant variables associated with innovation and technology in PC contexts (9). Based on these data, a set of questions was generated aiming to capture multiple dimensions of the theme. For the purpose of the questionnaire development, innovation in PC was operationalized as the introduction, adoption, or adaptation of new or significantly improved technologies, clinical practices, organizational processes, communication strategies, or educational approaches intended to improve the quality, accessibility, coordination, efficiency, or person-centeredness of care for patients and families. This working definition informed the selection of the survey domains and the development of items addressing technological integration, communication, education and training, symptom management, family support, care coordination, organizational conditions, and professional openness to change. However, no single operational definition of innovation was provided to participants. This approach was intentionally adopted to allow respondents to interpret innovation in line with their professional experience and organizational context, thereby capturing the diversity of meanings attributed to innovation in PC, and not limiting it to digital transformation. The questionnaire was then organized into eight sections: (1) demographic and professional background; (2) perceptions of innovation; (3) barriers to innovation; (4) facilitators of innovation; (5) knowledge and usage of innovation tools; (6) purposes/applications of digital health technologies; (7) openness to innovation; (8) future directions. The preliminary version of the questionnaire was reviewed by the HENKO-NET project partners, and the feedback received was incorporated into the final version prior to dissemination.

The full survey can be consulted in the Supplementary Material. Although the questionnaire was reviewed by the HENKO-NET consortium partners, no formal pilot testing or psychometric validation was undertaken prior to data collection, as the instrument was specifically developed for this exploratory study.

### 2.3 Participants and recruitment

The questionnaire was then disseminated through the HENKO-NET consortium via several online platforms, including the official HENKO-NET project website, institutional mailing lists, partner organizations, and social media platforms, reflecting the adoption of a convenience sampling strategy. Healthcare professionals currently working in PC were eligible to participate in the study. The questionnaire was accessible on the official website of the HENKO-NET project for response collection by PC professionals from January to July 2025.

### 2.4 Data analysis

#### 2.4.1 Quantitative analysis

Closed-ended and multiple-choice questions were analyzed quantitatively using Python (version 3.13.5) within the Anaconda distribution, with Visual Studio Code as the development environment. Data processing and visualization were conducted to support the interpretation of survey results. Descriptive statistics, including absolute frequencies and percentages, were used to summarize participants’ characteristics and survey responses. For multiple-response questions, each response option was analyzed independently according to the number of participants selecting that option. Given the exploratory nature of the study, the convenience sampling strategy, and the relatively small sample size, no inferential statistical analyses or subgroup comparisons were performed. As no missing data were identified, no imputation procedures were required, and the denominator for these analyses was the full analytical sample (*N* = 72).

#### 2.4.2 Qualitative analysis

On the other hand, open-ended questions were analyzed using Bardin’s content analysis framework, which comprises three main phases: (1) pre-analysis, including floating reading and definition of recording units; (2) material exploration, through inductive coding and development of thematic categories; and (3) treatment and interpretation of results, integrating emerging meanings. All 72 consenting participants were included in the qualitative component. Across the 14 open-ended fields, 710 textual entries were submitted and reviewed. Entries consisting solely of non-substantive responses, such as “No,” “No comments,” or a dash, were retained during screening but did not contribute to the development of substantive thematic categories, so no imputation for missing data was performed. The analysis was supported by ATLAS.ti software (version 24.1.1), which facilitated textual segmentation, code attribution, category grouping, and the construction of thematic networks, ensuring methodological rigor, transparency, and analytical traceability. The resulting categories emerged from an iterative process prioritizing fidelity to participants’ narratives and coherence with the study objectives. Category development was independently reviewed by two researchers, ensuring credibility and consistency in data interpretation. Any differences concerning code labels, code boundaries, the assignment of responses to thematic categories, and the interpretation of themes were discussed item by item. The coding framework and category definitions were refined through this process, and the final categorization was established by consensus. No formal inter-coder agreement coefficient was calculated.

#### 2.4.3 Integration of quantitative and qualitative findings

A convergent mixed-methods design was adopted, whereby quantitative and qualitative data were collected simultaneously using a single survey instrument, analyzed independently, and integrated during interpretation. Quantitative findings were used to describe patterns in participants’ responses, whereas qualitative findings provided explanatory depth by contextualizing and expanding these results. This integration enabled a more comprehensive understanding of healthcare professionals’ perceptions of innovation in PC.

### 2.5 Ethical considerations

Participation consisted of an anonymous, voluntary, non-interventional online questionnaire of healthcare professionals recruited through the professional contact networks of the HENKO-NET consortium. Before accessing the questionnaire, all participants received information regarding the objectives of the study and provided electronic informed consent prior to participation. No patient data, clinical records, biological samples, or identifiable personal information were collected. Data were collected and analyzed anonymously in accordance with ethical standards for research involving human participants. This study was reported in accordance with the Strengthening the Reporting of Observational Studies in Epidemiology (STROBE) guidelines, attached in Supplementary Material.

## 3 Results

A total of 72 responses were collected from healthcare professionals working in PC. Approximately 70% of participants were aged between 31 and 50 years. Regarding professional experience in PC, around 45% reported less than five years of experience, while 32% had been working in the field for more than ten years.

Responses were obtained from four countries, namely France, Spain, Portugal, and one response from Belgium. Therefore, the findings primarily reflect the perspectives of professionals from these regions and should not be interpreted as representative of all European settings. Participants represented a wide range of professional backgrounds and care settings. Medical doctors constituted the largest professional group (40.3%), followed by nurses (16.7%) and psychologists (13.8%). Most respondents worked in hospital-based services (51.4%) or in home care settings (31.9%). Detailed demographic information may be found in Table 1 (*N* = 72).

**Table 1:** Demographic and professional characteristics of participants (*N* = 72).

Demographic information	*n* (%)
Country of practice	
France	27 (37.5%)
Spain	27 (37.5%)
Portugal	17 (23.6%)
Belgium	1 (1.4%)
Gender	
Female	52 (72.2%)
Male	19 (26.4%)
Prefer not to say	1 (1.4%)
Age	
20–30 years old	6 (8.3%)
31–40 years old	27 (37.5%)
41–50 years old	23 (31.9%)
51–60 years old	14 (19.4%)
61 + years old	2 (2.8%)
Experience in palliative care	
0–5 years	32 (44.4%)
6–10 years	17 (23.6%)
11–15 years	13 (18.1%)
16–20 years	9 (12.5%)
21 + years	1 (1.4%)
Institution type	
Association	1 (1.4%)
Clinic	1 (1.4%)
Healthcare center	2 (2.8%)
Home care	23 (31.9%)
Hospital	37 (51.4%)
Hospital & home care	1 (1.4%)
Nursing home	1 (1.4%)
Social services	4 (5.6%)
Other	2 (2.8%)
Occupation	
Medical doctor	29 (40.3%)
Health executive	2 (2.8%)
Healthcare technician	5 (6.9%)
Nurse	12 (16.7%)
Occupational therapist	5 (6.9%)
Physiotherapist	1 (1.4%)
Psychologist	10 (13.9%)
Sculptor	1 (1.4%)
Senior health executive	1 (1.4%)
Social worker	5 (6.9%)
Other	1 (1.4%)

Data are presented as *n* (%). Percentages were calculated using the total analytical sample (*N* = 72). No demographic data were missing. Descriptive distribution of respondents by country of practice, gender, age group, years of experience in palliative care, institutional setting, and professional role.

Results are presented through the integration of quantitative survey findings and qualitative themes derived from participants’ open-ended responses. Quantitative findings describe the distribution of participants’ responses, while qualitative findings provide explanatory context and illustrate how healthcare professionals conceptualize and experience innovation in PC.

### 3.1 Perceptions of innovation

When asked to self-evaluate the level of innovation in their care setting, 4 participants (5.6%) rated it as very low, 31 (43.1%) as low, 29 (40.3%) as moderate, and 8 (11.1%) as high. Overall, 60 participants (83.3%) rated the level of innovation as low or moderate (*N* = 72; Figure 1). No dominant pattern emerged regarding the areas perceived as requiring greater innovation, since responses were relatively evenly distributed. The most frequently selected areas were “Training and Education” (*n* = 49), “Communication with patients” (*n* = 45), “Family support” (*n* = 40), “Technological integration” (*n* = 39), “Symptom control” (*n* = 33), and “Pain management” (*n* = 26). Qualitative analysis of responses provided under the open-ended option “Other” revealed that participants conceptualized innovation in PC beyond a purely technological perspective. Innovation was consistently framed to enhance care delivery, with a strong emphasis on preserving the human core of PC.

**Figure 1:**
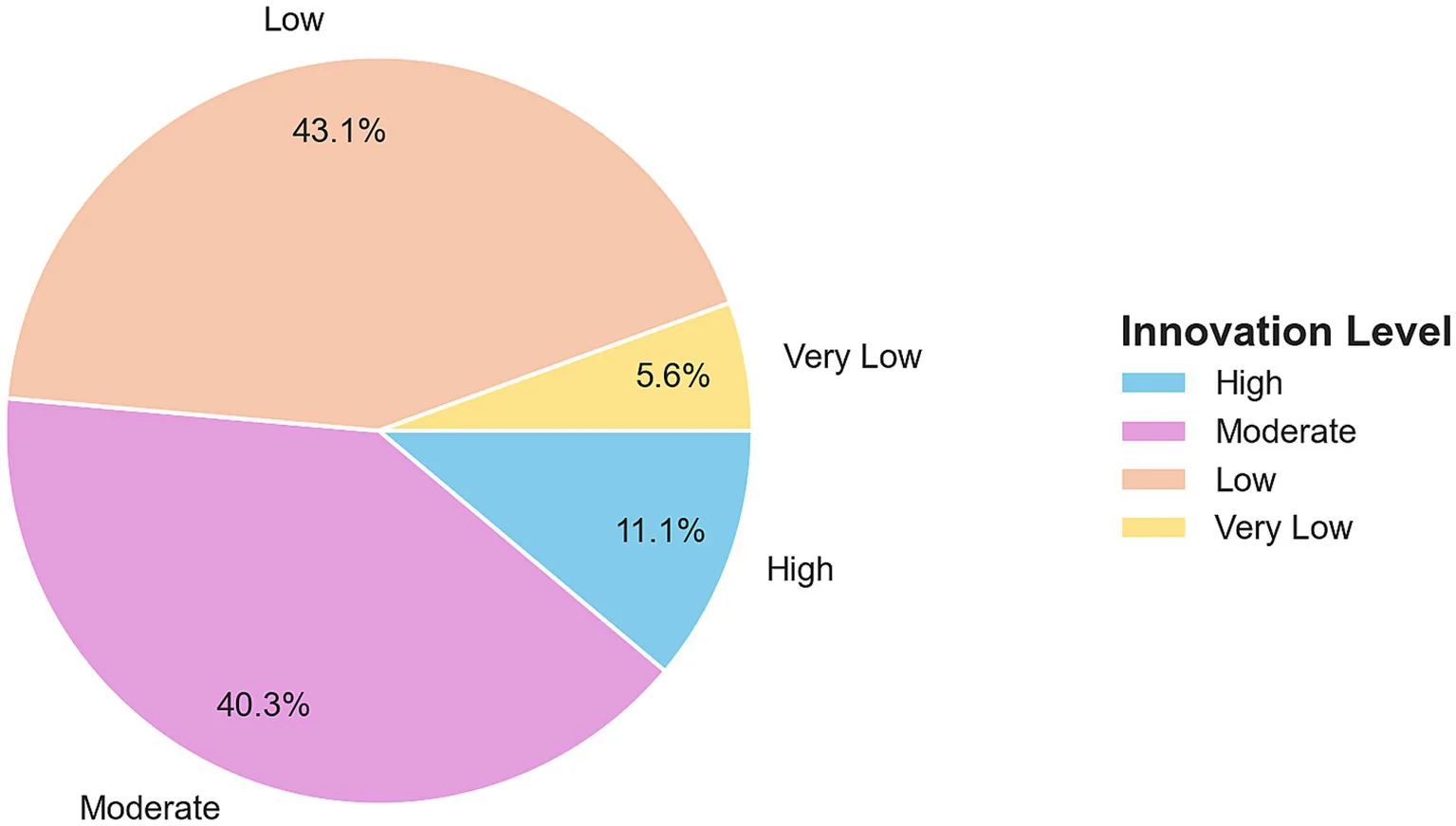
Pie chart showing the distribution of responses to the question: “How would you rate the level of innovation in your palliative care setting?”. Responses were recorded on a 5-point Likert scale ranging from “very low” to “very high”. Percentages are calculated using the full sample (*N* = 72) as the denominator. No responses were missing.

Thereafter, when asked about the types of innovations that could most improve patient care in palliative settings, participants further described a broad and interconnected set of priorities. Consistent with the working definition used to develop the questionnaire, innovation was not understood merely as technological advancement, but rather as a tool to foster proximity, reduce suffering, and improve integration across care processes. Participants’ open-ended responses encompassed technological, clinical, educational, relational, and organizational changes intended to improve care delivery while preserving the person-centered and human dimensions of PC.

#### 3.1.1 Supportive care technologies

##### 3.1.1.1 Enhancing proximity

Technology was frequently highlighted as a way to magnify the presence of care teams, particularly in home-based settings and situations of clinical vulnerability. Tools such as symptom telemonitoring, mobile applications, and teleconsultations were perceived as mechanisms to prevent avoidable suffering, enhance clinical safety, and provide reassurance to families.

*“Something that creates proximity and facilitates families’ adaptation to the caregiver role; tools that help identify patients’ needs in detail, allowing healthcare professionals to individualize and increasingly personalize their interventions.” (Participant ID6; translated from Portuguese)*

#### 3.1.2 Therapeutic communication

##### 3.1.2.1 Innovating the relational dimension of care

In line with the prior theme, participants emphasized communication as one of the most sensitive and critical components of PC, particularly in the context of complex decision-making and end-of-life discussions. Innovation in this domain was associated with the development of strategies and tools that support clear information-sharing, deep listening, and meaningful family involvement during difficult moments.

*“They can promote effective communication at the right time between patients and the team. Technology can help by providing tools that facilitate communication, such as telephone calls, text messages, chats, video, emails, and online portals.” (Participant ID1; translated from Portuguese)*

#### 3.1.3 Training and capacity building

##### 3.1.3.1 Enabling professionals to care better

Respondents consistently highlighted that the successful adoption of innovation depends on professionals feeling competent and confident. There was a strong call for continuous training in PC and ethical competencies, and the use of emerging technologies.

#### 3.1.4 Psychosocial and spiritual interventions

##### 3.1.4.1 Addressing invisible suffering

Participants stressed that suffering in PC is often emotional and existential. Desired innovations included psychosocial and spiritual interventions that help patients cope with fear, anxiety, loss, and meaning-making.

#### 3.1.5 Support for informal caregivers

##### 3.1.5.1 Caring for those who care

The need to support informal caregivers also emerged as a recurring theme. Participants emphasized innovations that provide practical guidance, emotional support, education, and rest, recognizing caregivers as central actors in home-based care whose well-being directly affects care continuity and quality.

#### 3.1.6 Management of complex symptoms

##### 3.1.6.1 Innovation to minimize pain

Despite advances in PC, respondents noted ongoing challenges in achieving rapid and effective symptom control. Innovation was associated with improved access to advanced therapies, particularly in the home setting, and more responsive approaches to complex symptom management.

#### 3.1.7 Policy and funding

##### 3.1.7.1 Enabling conditions for innovation

Finally, participants underscored that innovation cannot be sustained without adequate investment. Dedicated funding, expansion of care teams, and territorial equity were identified as fundamental conditions for meaningful innovation.

To sum up, participants articulated a vision of innovation grounded in two central goals: bringing care closer to patients through technology and communication, and empowering those who provide care through training, organizational support, and adequate resources.

### 3.2 Barriers to innovation

Regarding the perceived barriers to implementing innovative tools in PC, the most frequently selected obstacle was “Lack of funding” (*n* = 48), followed by “Resistance to change” (*n* = 44), “Lack of time” (*n* = 42), “Insufficient training” (*n* = 39), and “Regulatory Restrictions” (*n* = 16). Responses provided under the open-ended option “Other” reinforced that the main barriers to innovation in PC are not related to a lack of ideas or technological solutions, but rather to insufficient strategic investment, structural constraints, and cultural resistance. Participants consistently described innovation as failing not because it is undesired, but because the organizational and systemic context does not allow it to be sustained over time.

*“Staff shortage.” (Participant ID20; translated from French)*

*“Fear of losing the relational/human aspect or clinical skills … focusing solely on the screen and forgetting the patient.” (Participant ID33; translated from French)*

When asked to what extent they agreed with the statement “There is adequate support from management for innovation in my workplace”, 28 participants (38.9%) selected the neutral response category. Positive responses were reported by 27 participants (37.5%), comprising 20 (27.8%) who agreed and 7 (9.7%) who strongly agreed. Negative responses were reported by 17 participants (23.6%), comprising 10 (13.9%) who disagreed and 7 (9.7%) who strongly disagreed. Positive (agree/totally agree) and negative (disagree/totally disagree) perceptions were relatively balanced, suggesting variability across settings and a lack of consistent institutional backing for innovation (*N* = 72; Figure 2).

**Figure 2:**
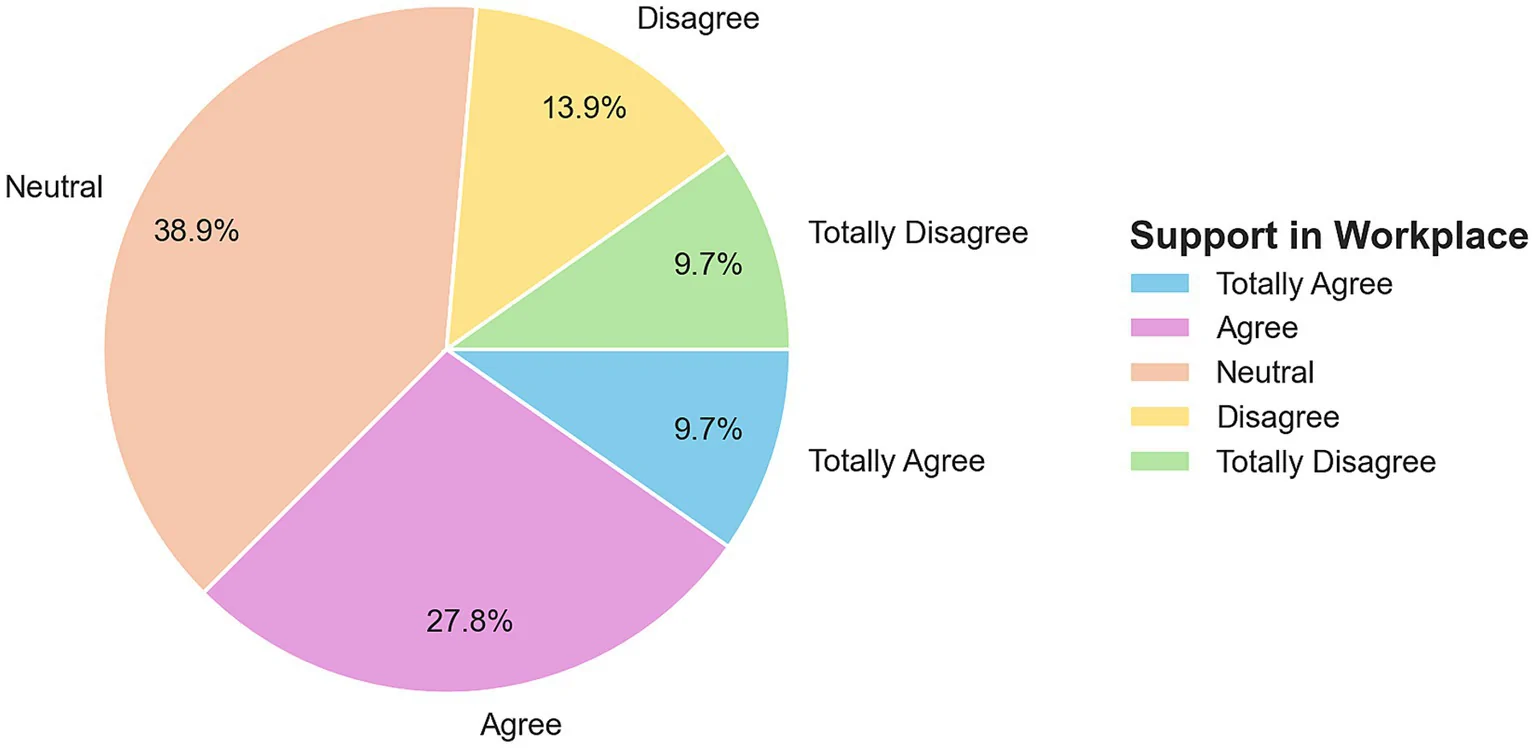
Pie chart depicting the distribution of responses to the statement: “There is adequate support from management for innovation in my workplace”. Responses were recorded on a 5-point Likert scale ranging from “totally disagree” to “totally agree”. Percentages are calculated using the full sample (*N* = 72) as the denominator. No responses were missing.

Participants were subsequently invited to describe any specific challenges encountered when attempting to introduce new practices or technologies into their work. Although responses to this question were generally shorter and more direct, they offered valuable insight into the concrete difficulties experienced on the frontline of care. Rather than abstract constraints, participants described handicaps encountered in everyday clinical practice. The challenges reported were largely consistent with those identified in previous questions and may be summarized as follows:

#### 3.2.1 Resistance and distrust within teams

Some participants identified colleagues as one of the main obstacles to implementation. Resistance to change, limited engagement with new routines, fear of losing established competencies, and generational or institutional cultural differences were frequently mentioned. This internal resistance was reported to undermine implementation processes, even when innovation was supported by part of the team.

#### 3.2.2 Lack of training and protected time for learning

Professionals emphasized that adopting new technologies or practices requires time for learning and adaptation, which is particularly difficult in contexts of high clinical workload. The absence of protected time for training, as well as situations in which innovation is introduced without prior capacity-building, were described as leading to frustration and, in some cases, early abandonment of the proposed changes.

#### 3.2.3 Insufficient material resources and organizational support

In line with the structural constraints described above, participants highlighted a lack of equipment, basic technological infrastructure, and technical or institutional support. Even when motivation and planning were present, innovation was often delayed or blocked by slow bureaucratic processes, rigid organizational structures, and lengthy project approval timelines.

*“Once funding has been secured, the hospital’s slow administrative and regulatory processes prevent new projects from being developed quickly.” (Participant ID35; translated from French)*

Respondents’ accounts suggest that innovation tends to fail not at the level of design or planning, but at the point of implementation, where care is actually delivered. Participants’ experiences indicate that successful innovation in PC requires adequately staffed and supported teams, flexible organizational structures, and an institutional culture that actively facilitates change.

### 3.3 Facilitators of innovation

Participants’ responses highlight that innovation in PC is primarily understood as a collective and relational process, rather than an individual or purely technical endeavor. “Collaboration & teamwork” emerged as the most prominent enabling factor (*n* = 59), followed by “Funding access” (*n* = 46), “Support policies” (*n* = 39), “Strong leadership” (*n* = 37), and “Patients & families’ engagement” (*n* = 28). Rather than introducing new ideas, responses in the open-ended “Other” option reinforced and expanded these priorities, pointing to the practical conditions that allow innovation to be sustained in everyday practice.

*“Strong leadership in managing the team that provides direct patient care.” (Participant ID8; translated from Portuguese)*

In line with these findings, most participants (52.8%) reported that continuing education and professional development are encouraged or supported in their workplace, suggesting a generally favorable environment for professional growth, albeit with notable variability across settings (*N* = 72; Figure 3).

**Figure 3:**
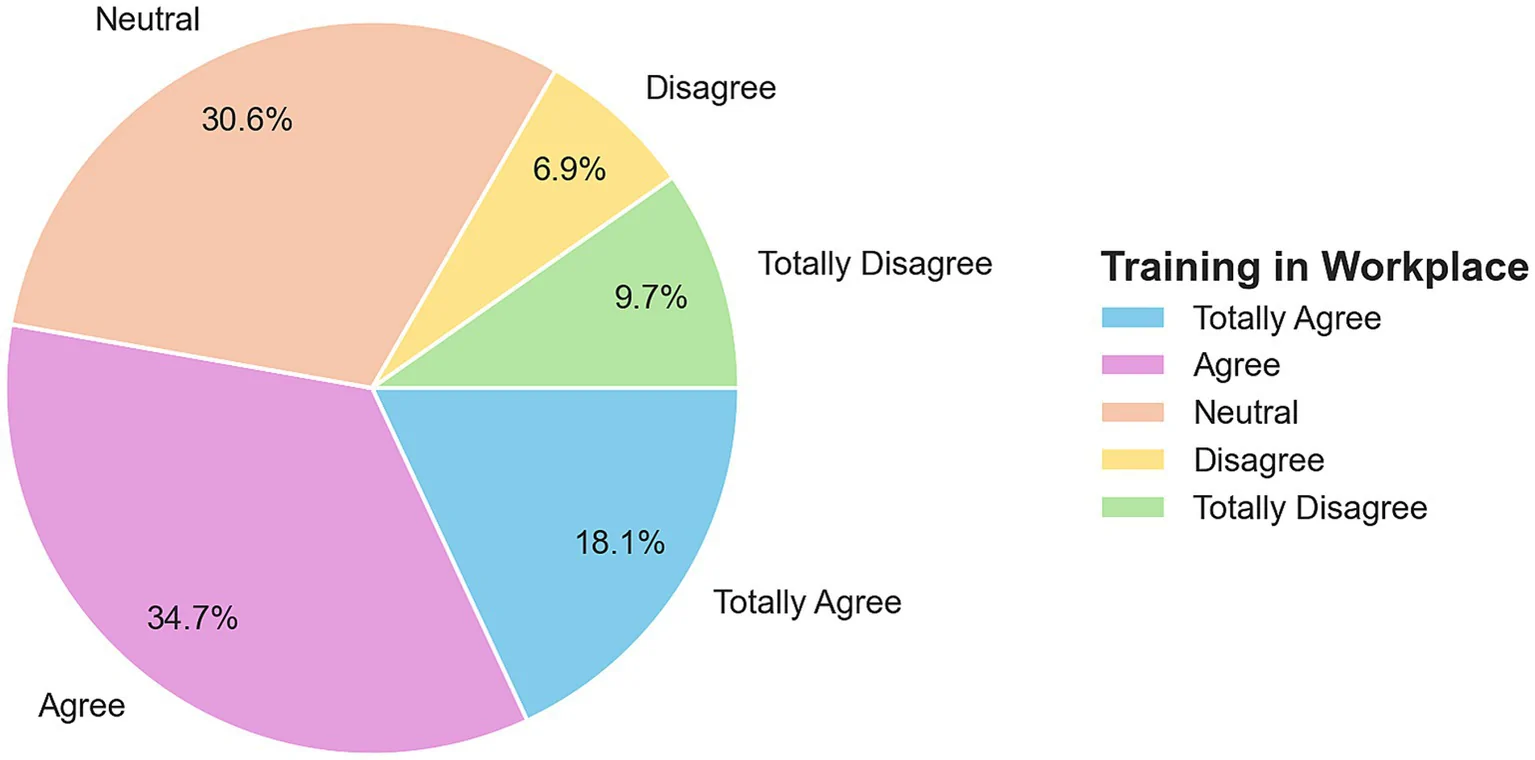
Pie chart representing the distribution of responses to the statement: “continuing education and professional development are encouraged and supported in my workplace”. Responses were recorded on a 5-point Likert scale ranging from “totally disagree” to “totally agree”. Percentages are calculated using the full sample (*N* = 72) as the denominator. No responses were missing.

When asked to share examples of successful innovations implemented in their PC settings, only a limited number of participants provided detailed responses. Nevertheless, the examples reported offer valuable insight into feasible and effective innovation practices already in place, demonstrating how innovation can be operationalized in real-world PC contexts.

#### 3.3.1 Communication technologies and tele-support

Compliant with needs previously identified, several participants described initiatives aimed at improving communication between healthcare teams, patients, and families. These included teleconsultations during clinical deterioration or when in-person visits were not feasible, as well as digital communication systems supporting continuity of care. Such tools were valued for their capacity to reduce physical distance, anticipate needs, and strengthen support in home-based care.

#### 3.3.2 Structured protocols and new clinical practices

Other responses referred to the implementation of standardized clinical protocols, particularly for symptom control and transitions of care. These initiatives were perceived as enhancing clinical safety, promoting consistency in care delivery, and supporting shared decision-making within multidisciplinary teams.

*“For the past two years, we have implemented the RUN-PC Tool methodology to triage patients on the waiting list.” (Participant ID2; translated from Portuguese)*

#### 3.3.3 Psychosocial innovation and meaningful activities

Participants also reported practices focused on emotional well-being and quality of life, including activities designed to promote comfort, connection, and a sense of normality. Although often simple, these interventions were described as highly meaningful and capable of positively transforming patients’ everyday experiences.

These examples demonstrate that innovation is possible when enabling conditions are present, even if it is often implemented on a localized or small-scale basis. They also indicate that professionals are capable of creativity and adaptability despite resource constraints, reinforcing the alignment between existing innovative practices and the priorities expressed across previous sections.

### 3.4 Knowledge and usage of innovation tools

A direct comparison between the number of innovation tools known and those currently used in participants’ settings showed a clear gap between awareness and implementation. While healthcare professionals reported being familiar with a wide range of recent technological solutions, these tools were not routinely integrated into their practice. This discrepancy is well illustrated in Figure 4. Among the tools that were reported as being available, more than 90% were used on a daily basis.

**Figure 4:**
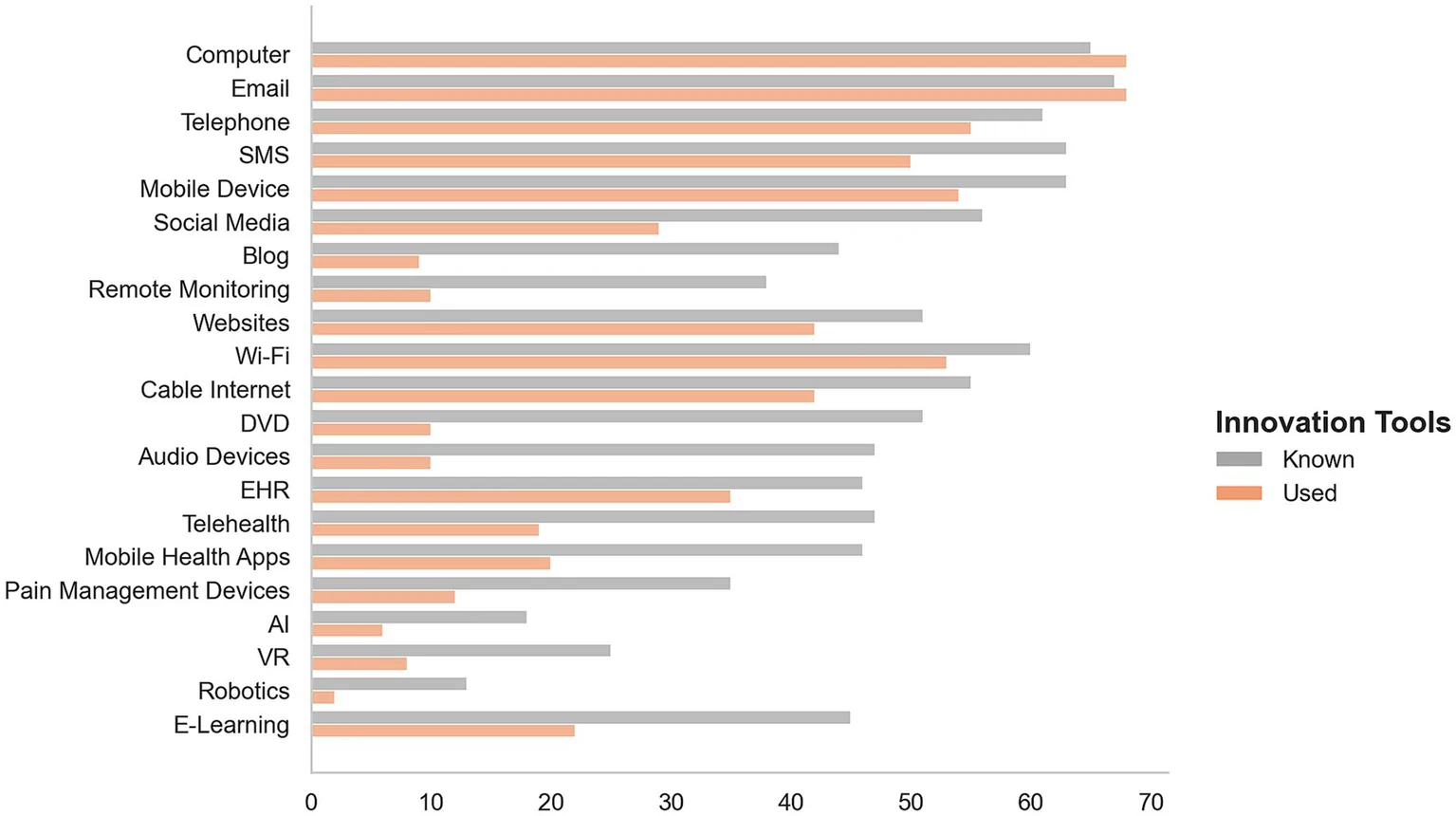
Bar chart comparing the proportion of participants reporting familiarity with specific digital technologies (“known”) and those reporting their active use in clinical practice (“used”). Participants could select multiple technologies; therefore, categories are not mutually exclusive and frequencies cannot be summed to obtain the total number of participants.

When asked whether the use of innovative tools had improved the quality of PC in their setting, approximately half of the participants reported a positive perception, while 35% expressed a neutral position, and the remaining participants disagreed (*N* = 72; Figure 5).

**Figure 5:**
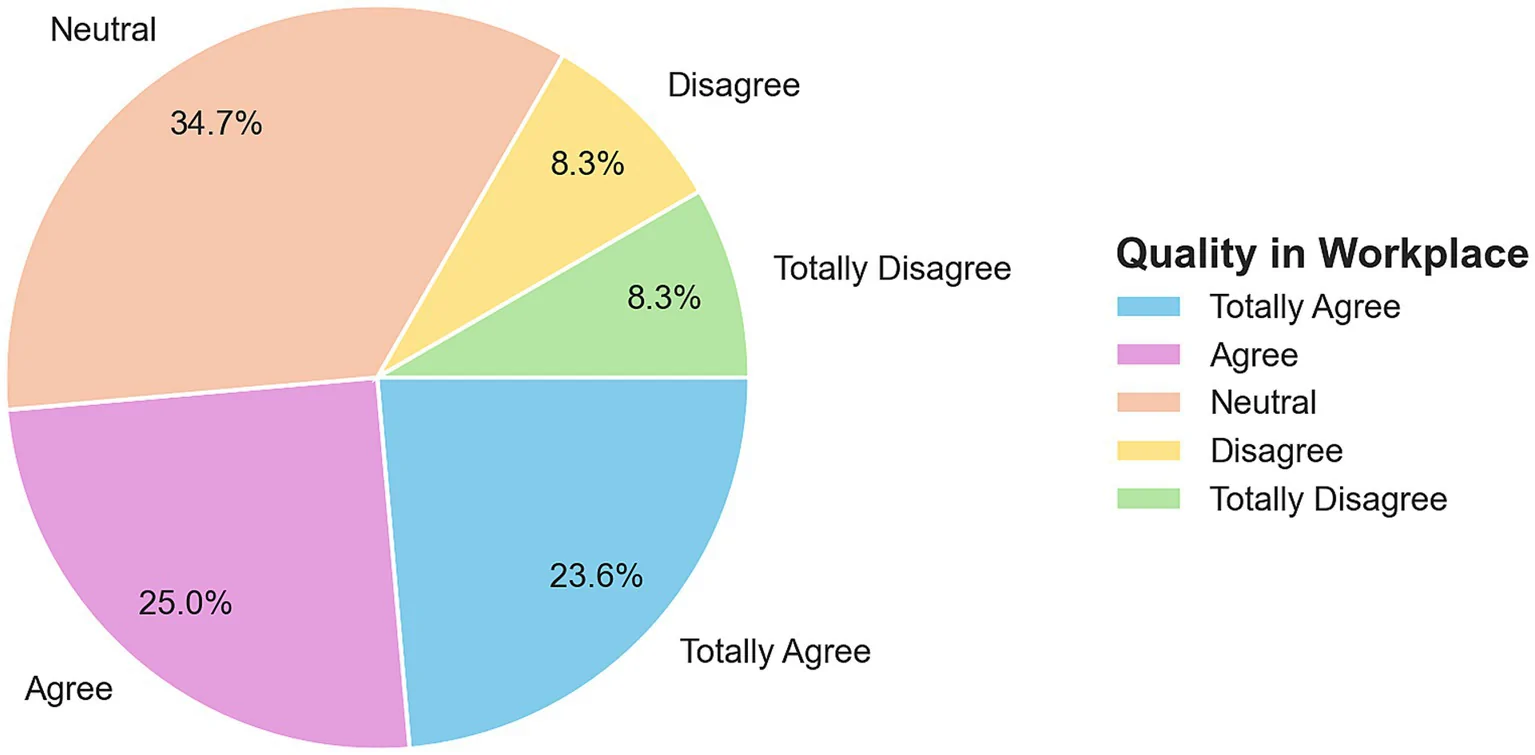
Pie chart showing the distribution of responses to the statement: “The use of innovation tools has improved the quality of palliative care provided in my setting”. Responses were recorded on a 5-point Likert scale ranging from “totally disagree” to “totally agree”. Percentages are calculated using the full sample (*N* = 72) as the denominator. No responses were missing.

Responses to the question “Are there any innovation tools that you would like to use but currently do not have access to?” identified several unmet technological and organizational needs, particularly relating to home-based care and coordination across services. Participants consistently described situations in which relevant solutions were known but could not be accessed or implemented in practice.

#### 3.4.1 Telemonitoring and teleconsultation tools

Earlier pinpointed as a domain needing improvement, participants frequently referred that remote symptom monitoring devices, rapid-response teleconsultation tools, and digital platforms enabling real-time information exchange would be valuable within care teams. These technologies were perceived as essential for anticipating clinical decline, reducing avoidable hospital visits, and enhancing safety and reassurance in home-based PC.

#### 3.4.2 Interoperable digital information systems

A recurring concern was the lack of interoperability between existing digital systems. Participants described fragmented information infrastructures that hinder continuity of care, generate duplicated documentation, and increase the risk of information loss. Access to shared electronic health records and interoperable platforms across levels of care was identified as a priority to support coordinated and timely clinical decision-making.

#### 3.4.3 Clinical support equipment and decision-support tools

Several responses highlighted limited access to appropriate clinical equipment, decision-support technologies, and essential materials for home-based PC. These constraints were described as restricting professional autonomy and reducing the ability to respond effectively to complex symptom management in the home setting.

Taken together, participants’ responses indicated that the most valued innovation tools are those that support clinical presence, timely intervention, and anticipation of patient needs, rather than those that add technological complexity or administrative burden.

### 3.5 Purposes/applications of digital health technologies

Digital health technologies currently in use were most frequently applied to “Medical records” (*n* = 44), “Communication with patient” (*n* = 39), “Care coordination” (*n* = 37), “Medicine management” (*n* = 35), “Continuous training” (*n* = 32), “Symptoms management (*n*=25), “Teleconsultation” (*n* = 23), and “Decision support” (*n* = 18).

Participants’ responses regarding additional purposes/applications for which they would like to use digital health technologies, but which are not currently available, were generally brief yet coherent in their focus. Answers were cohesive with the precedent, with a clear preference for tools that strengthen communication and enable continuous follow-up, particularly in home-based care contexts, while indicating that current technological use falls short of these expectations.

#### 3.5.1 Enhanced communication with patients and families

Once again, participants expressed the need for digital solutions that allow rapid and reliable contact with healthcare teams. Digital technologies were described as a means of providing reassurance and reducing anxiety, particularly for families supporting patients at home.

*“Improve communication with both patients and families, as well as among professionals.” (Participant ID64; translated from Spanish)*

#### 3.5.2 Remote monitoring of symptoms and early warning signs

Several respondents anew emphasized the value of systems capable of collecting real-time clinical data, generating alerts for clinical worsening, and supporting early intervention.

#### 3.5.3 Education and empowerment of informal caregivers

Acknowledging the central role of families in PC, participants highlighted the need for digital tools that support caregiver education and confidence. Suggested resources included instructional videos, decision-support materials, and digitally mediated psychological support aimed at increasing caregivers’ preparedness and reducing emotional burden.

#### 3.5.4 Interprofessional and interinstitutional coordination

The lack of interoperability between services emerged again as a hot topic. Participants advocated for shared digital platforms, accessible and integrated clinical records, and coordinated care plans, emphasizing that digital health technologies should facilitate network-based care rather than perpetuate fragmented practices.

Despite the reported gaps in availability and scope, most participants considered the digital tools currently in use to be effective (56.9%) or very effective (15.3%) for the purposes they were designed to serve, as shown in Figure 6 (*N* = 72).

**Figure 6:**
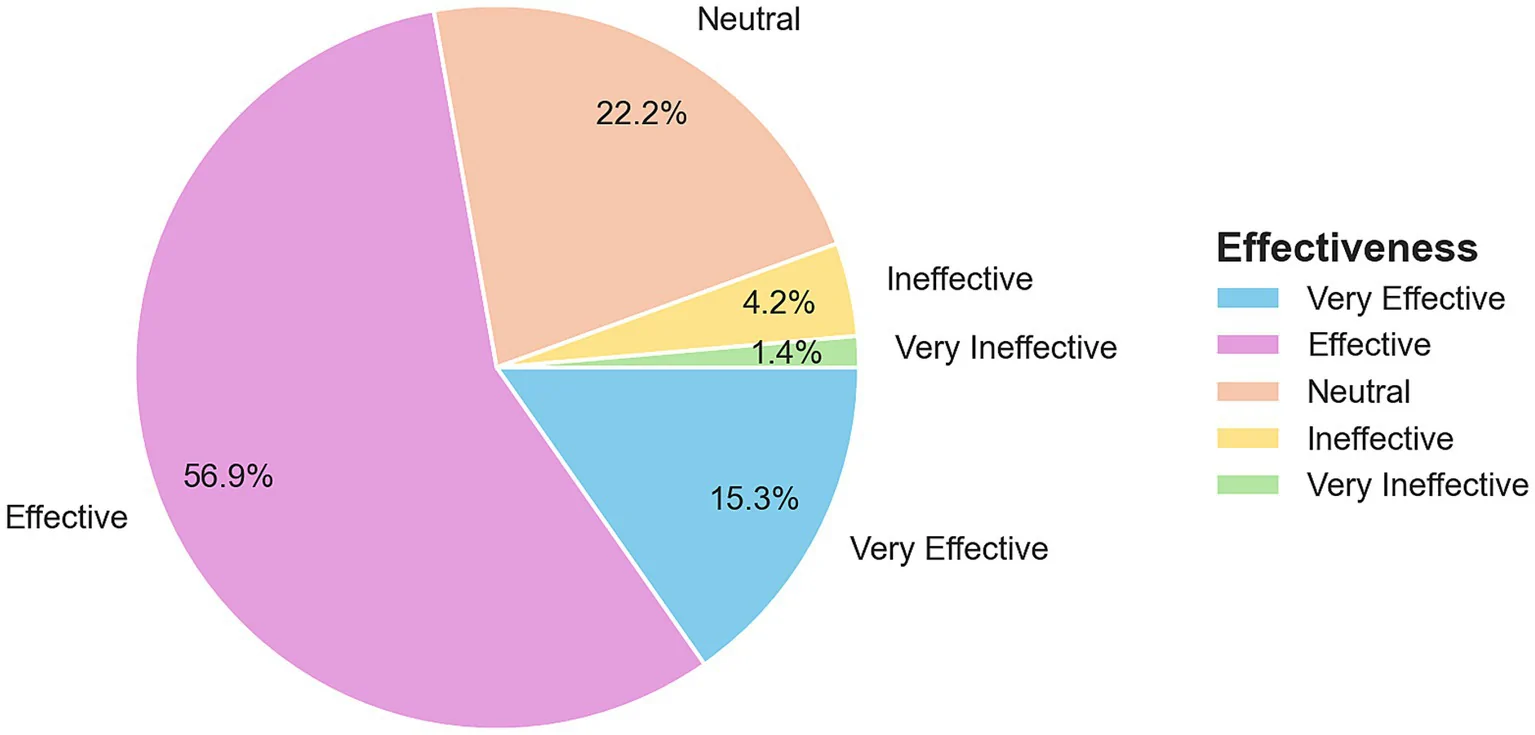
Pie chart depicting the distribution of responses to the question “How do you rate the effectiveness of digital health technologies/applications for the purposes mentioned above?”. Responses were recorded on a 5-point Likert scale ranging from “very ineffective” to “very effective”. Percentages are calculated using the full sample (*N* = 72) as the denominator. No responses were missing.

### 3.6 Openness to innovation

Alongside the identification of unmet needs related to innovation tools, participants expressed a strong willingness to integrate new technologies into their PC practice. In fact, as presented in Figure 7 (*N* = 72), more than 90% of participants reported being open to integrating innovation tools into their PC practice. The factors most likely to influence their willingness to adopt a technology included “User friendliness” (*n* = 57), “Proven effectiveness” (*n* = 50), “Training & support availability” (*n* = 43), “Patients’ preferences” (*n* = 38), “Institutional support” (*n* = 38), “Costs” (*n* = 23), and “Colleagues recommendation” (*n* = 16). In parallel, over 90% of participants expressed confidence in their ability to learn and use innovation tools.

**Figure 7:**
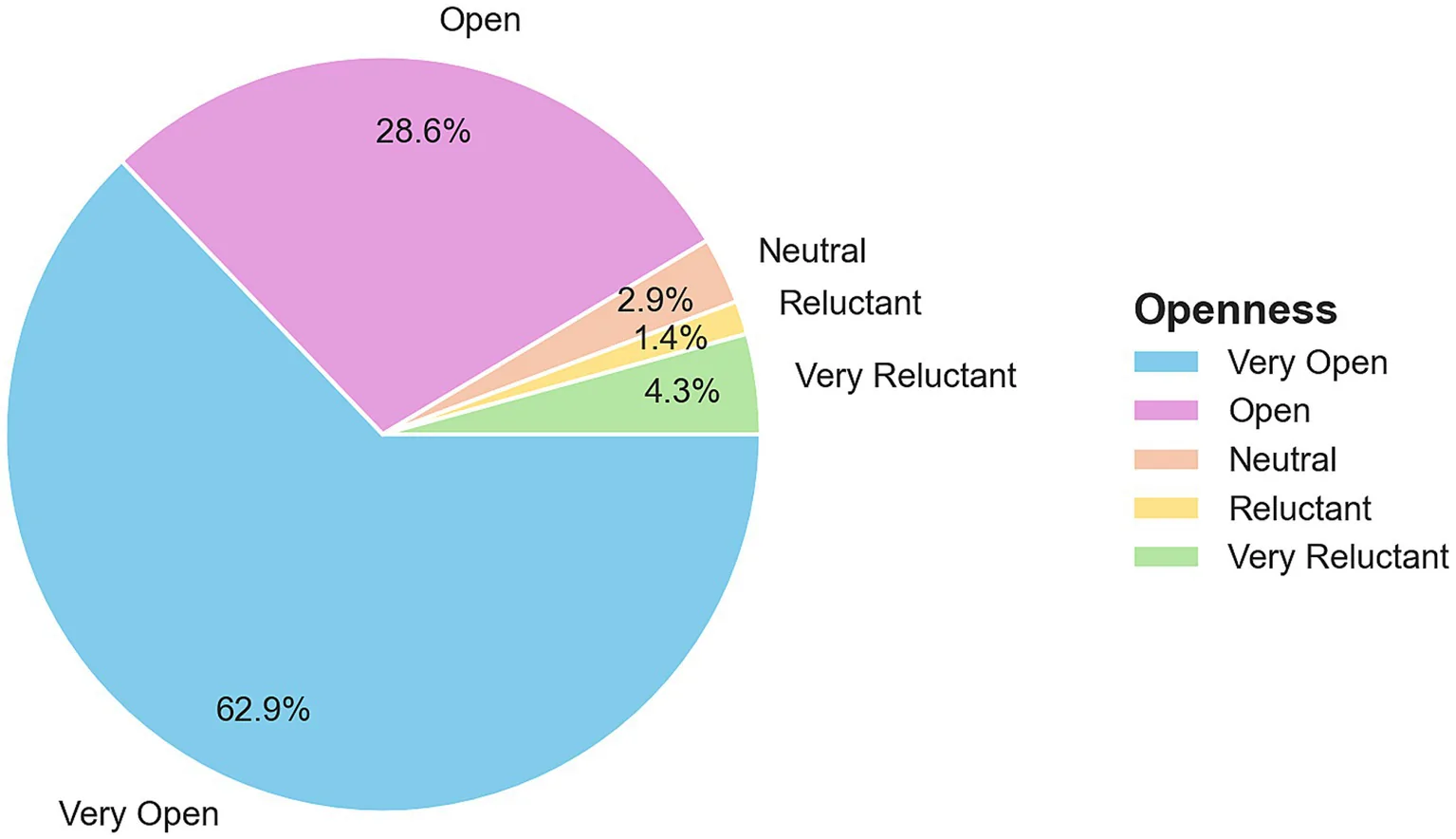
Pie chart exhibiting the distribution of responses to the question “How open are you to integrating new innovation tools into your palliative care practice?”. Responses were recorded on a 5-point Likert scale ranging from “very reluctant” to “very open”. Percentages are calculated using the full sample (*N* = 72) as the denominator. No responses were missing.

When asked about the support or resources needed to feel more comfortable adopting innovation tools, participants provided concise and convergent responses. These focused on practical conditions required to translate openness into sustained use, reflecting a clear awareness of the gaps that currently limit implementation.

#### 3.6.1 Accessible training and allocated time for learning

The most frequently identified need was continuous and targeted training, accompanied by institutional allocation of assigned time for learning.

*“Continuous training and allocated time.” (Participant ID13; translated from Portuguese)*

#### 3.6.2 Technical support and professional follow-up

Respondents highlighted the importance of not being left alone when facing technical challenges. The presence of responsive IT teams, dedicated technical support, and accessible helpdesk services was perceived as essential for developing the “know-how” and ensuring continuity in the use of innovation tools.

*“Better technical support to free up multidisciplinary human time.” (Participant ID42; translated from French)*

#### 3.6.3 Adequate material and technological resources

Several participants pointed to concrete infrastructural needs, including access to appropriate equipment, devices suitable for home-based care, reliable connectivity, and robust digital systems. The absence of these basic resources was described as a practical limitation to the operationalization of innovation.

#### 3.6.4 Organizational encouragement and legitimacy

Finally, participants underlined the need for explicit institutional endorsement, including leadership support, recognition of the effort required to innovate, and realistic expectations regarding implementation. Organizational legitimacy was viewed as central to transforming individual willingness into routine practice.

Collectively, these responses depict professionals who are highly receptive to innovation, if learning opportunities, technical support, material resources, and organizational backing are aligned with expectations for change.

### 3.7 Future directions as perceived by professionals

Responses to the question on which key steps are needed to bolster innovation in PC were richer and more reflective than in previous items, often resembling a strategic journey outlined by the professionals themselves. Participants consistently identified multiple areas requiring change or significant improvements to support innovation implementation in PC:

#### 3.7.1 Advancing training and professional development

Capacity building was consistently identified as the starting point for innovation. Innovation was perceived as intrinsically linked to professional competence.

#### 3.7.2 Cultural change and openness to innovation

Respondents highlighted the need for a cultural shift within organizations. This includes openness to new ideas, reducing fear that innovation may undermine the human dimension of care, and fostering environments that value learning, experimentation, and reflective practice. As well as a stronger political and institutional commitment to PC, including the expansion of teams, sustained funding, integration of technological solutions into routine services, and equitable access across regions.

#### 3.7.3 Service organization focused on continuity of care

The rearrangement of care services emerged as a critical step, with professionals advocating for more versatile systems, permanent home-based support, effective networking between levels of care, and a reduction in bureaucratic barriers.

#### 3.7.4 Effective communication: care as a relational encounter

Finally, innovation was closely linked to how care is communicated and co-constructed. Participants stressed the importance of structured communication, shared decision-making, and health literacy support for patients.

These responses depict innovation in PC as a multidimensional process, integrating education, culture, resources, organization, and communication, while remaining centered in the human essence of care.

Beyond identifying concrete steps for fostering innovation, participants were also asked to reflect on how they envision the future of PC. Their portrayals of the future of PC were shaped by both hope and critical realism. Innovation was widely perceived as inevitable and necessary; however, its impact on health systems’ ability to integrate, sustain, and humanize technological and organizational change was seen as uncertain. In general, the future of PC was envisioned as more technological, integrated, and alert to existential suffering, with human dignity at its core.

#### 3.7.5 A more technological future: innovation as an extended presence

Professionals acknowledged that digitalization is no longer optional but an irreversible path. They anticipated a future in which telemonitoring enables early identification of crises, teleconsultations reduce burdensome hospital visits, and real-time data sharing enhances clinical safety and continuity of care, particularly in home-based settings. Technology was framed as a means of extending presence rather than replacing it.

#### 3.7.6 A more integrated future: innovation in service of continuity

Participants emphasized the need for greater integration across care settings, envisioning smoother transitions between hospitals, primary care, and specialized palliative teams. Innovation was seen as effective only if it supports collaboration among professionals and institutions, addressing the current isolation that contributes to avoidable suffering.

#### 3.7.7 A more human future: innovation reinforcing care relationships

Despite strong endorsement of technological progress, respondents consistently stressed that innovation must strengthen, rather than undermine, the human dimension of care. Communication, emotional and spiritual support, and respect for patients’ individual rhythms were highlighted as non-negotiable.

*“Innovation should be encouraged in this field, but always with balance.” (Participant ID5; translated from Portuguese)*

#### 3.7.8 A future constrained by present challenges

Alongside optimism, some participants expressed skepticism regarding the pace and feasibility of innovation, citing persistent hurdles such as insufficient investment, limited public awareness of PC, staff shortages, and institutional resistance to change. These concerns underscore that innovation, although desired, is not yet guaranteed.

#### 3.7.9 A more visible and recognized future for palliative care

Lastly, respondents called for greater societal recognition of PC, upholding earlier integration, equitable access, and public demystification. Innovation was linked to the affirmation of PC as a central response to serious illness, rather than a late or marginal intervention.

Finally, the closing open-ended questions invited participants to share any additional comments or suggestions regarding innovation in PC. These elicited fewer substantive responses, as several participants answered briefly (e.g., “No,” “No comments”). Nonetheless, the contributions with significant content offered reflective insights that reproduce the tension between the desire for change and the awareness of persistent constraints. Participants emphasized that innovation must remain firmly person-centered and aligned with the fundamental principles of PC, and that without sustained investment, innovation cannot be maintained. Protected time for training and clinical reflection was called for, as well as institutional support to safely test and adopt new practices. Lastly, respondents, once again, stressed that innovation is considered meaningful only if it brings people closer rather than further apart.

*“Maintain humanity; do not make care cold and insensitive.” (Participant ID11; translated from Portuguese)*

Across these final reflections, participants articulated a vision of innovation grounded not only in technological and organizational change but in the commitment to preserve dignity, relational care, and proximity at the end of life. To integrate the quantitative and qualitative findings, Figure 8 presents an author-developed interpretative framework encompassing participants’ perceived drivers, constraints, and future-oriented dimensions of innovation in PC, where person-centered and relational care is positioned as a cross-cutting central principle.

**Figure 8:**
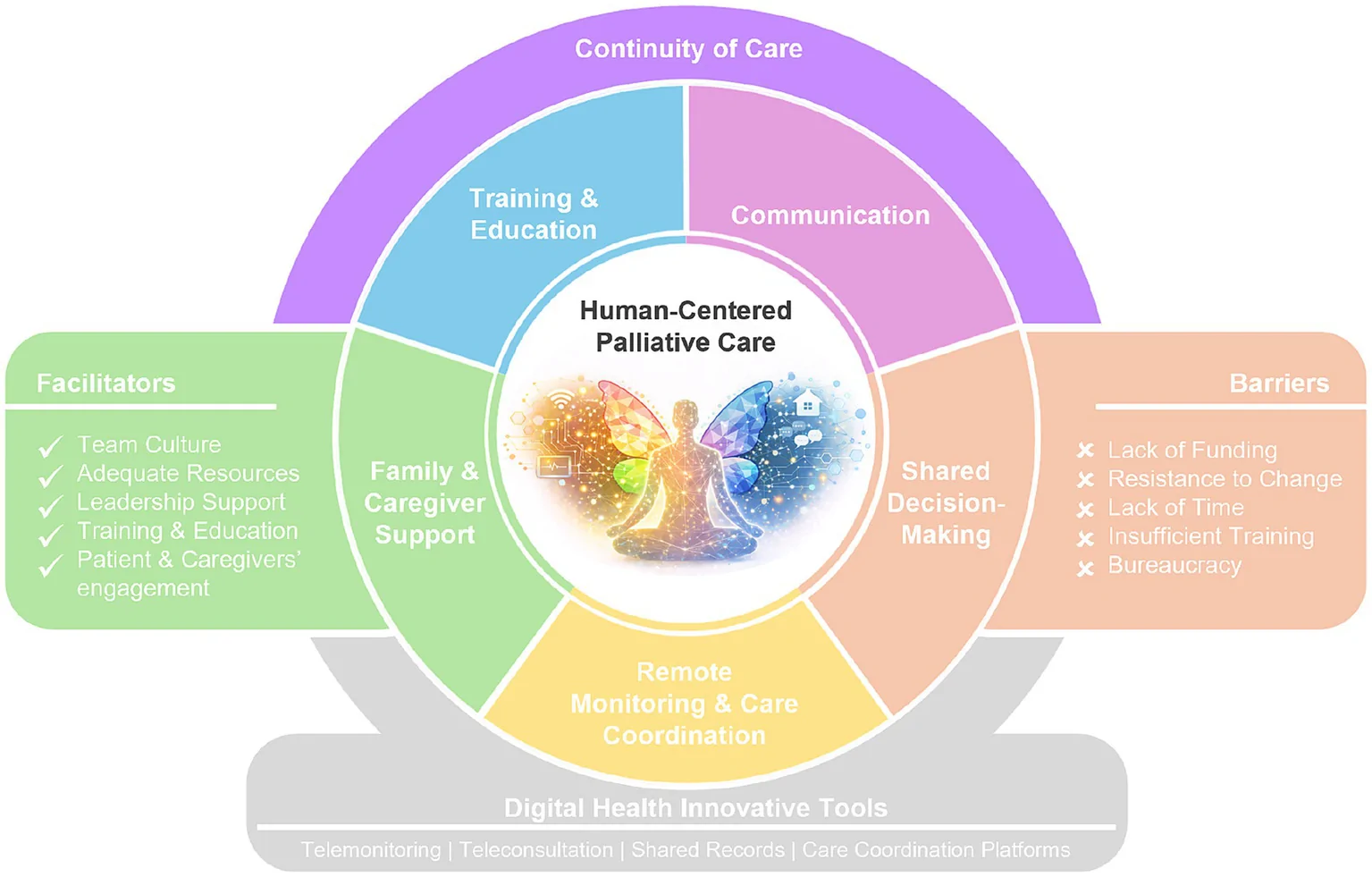
Human-centered interpretative framework of innovation in palliative care derived from cross-contextual professional perspectives. The model illustrates the interrelationship between structural determinants (policy, organizational readiness, infrastructure), professional attitudes, implementation barriers and facilitators, and the role of digital tools in supporting relational and continuity-based care.

## 4 Discussion

This study offers a territorial and cross-contextual analysis of healthcare professionals’ attitudes toward innovation in PC, revealing a multidimensional landscape characterized by high openness, conceptual breadth, and structural constraint. The integration of quantitative and qualitative findings enabled a more comprehensive interpretation of healthcare professionals’ perspectives by combining descriptive patterns with contextual insights derived from participants’ narratives. The results highlight a central paradox: innovation is widely desired and ethically reframed by professionals, yet its implementation remains constrained by systemic and organizational fragilities.

Interestingly, more than 80% of participants rated the level of innovation in their care setting as low or moderate, suggesting that PC innovation across respondents’ contexts remains at an intermediate stage of maturity. This perception persists despite increasing policy emphasis on digital transformation across Europe (10). When asked where innovation was needed most, answers were spread across different areas, and professionals did not associate innovation exclusively with technology. Instead, they articulate a broad innovation agenda that includes relational, educational, organizational, and clinical domains. This heterogeneity may limit the comparability of quantitative responses but also reflects the multidimensional nature of innovation in PC. These findings are aligned with holistic PC frameworks, which emphasize multidimensional suffering and interdisciplinary responses (11). The strong focus on communication and caregiver support underscores the need for innovation in this field to extend beyond efficiency-driven digitalization and address relational continuity and psychosocial complexity.

In addition to identifying priority areas, qualitative findings consistently framed innovation as a tool to “bring care closer” rather than to modernize for its own sake. Tools such as telemonitoring and teleconsultation were valued for enhancing safety and anticipation in home-based care, but only to the extent that they strengthened relational presence. This interpretation is supported by prior research demonstrating that tele-palliative interventions are most effective when embedded in patient-centered communication strategies rather than implemented as isolated technical solutions (3, 12, 13).

Moreover, participants’ emphasis on dignity, proximity, and existential support highlights the ethical specificity of innovation in end-of-life contexts. The findings corroborate that, in contrast to other clinical domains where innovation may prioritize speed or automation, PC innovation must reconcile digital transformation with preservation of relational integrity. This reinforces arguments from digital health experts that human-centered design and workflow compatibility are essential for sustainable implementation (14, 15). However, although the human dimension of care was frequently invoked, this study did not explicitly explore the ethical tensions associated with digital health technologies in PC. Future research should engage more directly with bioethical frameworks to examine how dignity-preserving and relationally grounded innovation can be operationalized in practice.

Furthermore, a key finding of this study is the evident discrepancy between knowledge of innovation tools and their actual integration into practice. While professionals reported familiarity with a wide range of digital solutions, implementation remained uneven. Among tools that were available, over 90% were reportedly used daily, suggesting that once structural access is ensured, adoption is not a major obstacle. However, the overall gap between awareness and access indicates systemic bottlenecks in the translation of innovation into routine care. Similar gaps have been documented in digital health research, where non-adoption and limited scale-up are frequently linked to contextual misalignment rather than technological inadequacy (16). Importantly, this study captures self-reported attitudes and perceptions rather than observed behaviors. The gap between declared knowledge and actual integration of innovation tools into clinical practice remains an important area for future research, particularly through observational or implementation-focused designs.

In line with this interpretation, other studies have shown that structural readiness, rather than professional resistance, is often the limiting factor in healthcare innovation (14, 17). In our study, the most frequently cited barriers reflected multilevel determinants spanning macro (policy and funding), meso (organizational culture and leadership), and micro (individual competence and workload) dimensions.

Importantly, resistance to change was not described primarily as ideological opposition, but rather as cultural inertia, fear of increased workload, or generational differences. This distinction is critical. Research on organizational change suggests that what is often labeled as “resistance” may reflect perceived lack of resources, insufficient psychological safety, or uncertainty about role adaptation rather than conceptual disagreement with innovation itself (5, 18). The present results therefore suggest that participating professionals are not rejecting innovation per se, but require legitimacy, training, and institutional endorsement to integrate change sustainably.

Taken together, our findings strongly indicate that innovation failure in participants’ settings is linked to structural fragility rather than conceptual resistance. Participants repeatedly described insufficient human resources, workforce overload, bureaucratic delays, and fragmented digital infrastructures as implementation barriers. These constraints are consistent with broader European evidence documenting underinvestment and variability in PC services (10, 19).

Within complex adaptive systems, innovation should not be imposed linearly; it must emerge from aligned incentives, leadership support, infrastructure, and professional capacity (20). Introducing innovation into overburdened teams without protected time or training risks generating frustration and implementation fatigue. The high percentage of participants expressing openness to innovation alongside concerns about structural support underscores this paradox. Nevertheless, no single operational definition of innovation was provided in the questionnaire, allowing participants to interpret the concept of innovation according to their own professional and contextual understanding, which might have biased this result.

Alongside barriers, several facilitators were also pinpointed. The findings were consistent with the Consolidated Framework for Implementation Research and Normalization Process Theory, which identify leadership engagement, resource allocation, and cultural legitimacy as central determinants of sustained innovation (17, 21). Moreover, continuing education was reportedly encouraged in most settings, suggesting a favorable foundation for capacity building. However, participants emphasized once more the need for allocated time and technical support. Thus, validating that innovation is not simply a matter of willingness, but of structured enablement.

Following this connection, Figure 8 presents an interpretative framework developed to synthesize the main findings of this exploratory study by integrating the descriptive quantitative results with the thematic analysis of participants’ open-ended responses. The framework provides a study-specific visual representation of areas of convergence and complementarity between the two data sources, with human-centered palliative care placed at its core. Surrounding this central perspective are the main domains identified as relevant to care delivery and as potential areas for innovation. The framework also highlights, on each side, the main facilitators and barriers reported by participants, reflecting the organizational and structural conditions that may enable or constrain innovation. Continuity of care is positioned as an overarching outcome, reflecting the recurring emphasis placed by participants on maintaining connected, coordinated, and person-centered care across settings. At the base, digital health technologies are presented as enabling tools that can support these domains and elevate continuity of care. Overall, the framework illustrates how innovation in PC may emerge from the interaction between technological opportunities, organizational conditions, and the relational dimensions of care.

Additionally, the results conjecture an influence of territorial context. While the study was not designed for comparative national analysis, responses from France, Spain, and Portugal revealed variability in perceived management support. For instance, although most participants answered a neutral response, Spanish participants reported a higher support from management for innovation in their workplace (55.5% agree or totally agree) than Portuguese and French participants (29.4 and 22.2%, respectively). In fact, European health systems substantially differ in funding models, digital interoperability, and service organization (22). Nonetheless, given the relatively small country-specific samples, unequal representation, and convenience sampling strategy, the observed variability cannot be attributed to national context. These patterns should thus be interpreted as descriptive observations within the study sample rather than as evidence of differences between countries.

This underlines a limitation of our study. Although it benefits from its cross-national scope and mixed-methods design, the sample size and unequal distribution across participating settings, including only one respondent from Belgium, limit generalizability, and robust cross-comparative analysis could not be performed. This also precluded additional powered subgroup analyses, such as comparisons by professional category, care setting, or years of experience. Future research could explore longitudinal implementation trajectories or investigate whether responses vary according to country, professional role, care setting, and years of experience. Other limitations include sample bias. The study relied on a convenience sample recruited through the HENKO-NET network, which may have introduced self-selection bias and resulted in a sample with a higher baseline interest in innovation and higher digital literacy than the wider PC workforce. The results should therefore be interpreted as exploratory and should not be considered representative of all PC professionals across Europe. Moreover, the predominance of medical doctors and hospital-based professionals, and the underrepresentation of certain professional groups, particularly frontline care assistants involved in domiciliary care, may have limited the comprehensiveness of the perspectives captured, particularly in relation to organizational structures and access to digital resources. Lastly, an additional limitation concerns the questionnaire itself. Although the instrument was developed following a comprehensive review of the literature and revised by the HENKO-NET consortium partners before dissemination, no formal pilot testing or psychometric validation was undertaken. Consequently, future research should evaluate its measurement properties, including validity and reliability, in larger and more diverse samples.

Reflecting upon the future of PC, participants expressed both optimism and caution. While many anticipate a more technological and integrated future, concerns about depersonalization and resource scarcity persist. This duality underscores the need for ethical safeguards in digital transformation. Following this perspective, healthcare professionals believe innovation strategies in PC must explicitly integrate relational quality indicators, caregiver burden metrics, and dignity-preserving practices. Technology should function as an extension of presence rather than a replacement (23), reinforcing, once more, the importance of human-centered digitalization in sensitive care domains. This also aligns with broader bioethical discussions in PC, which emphasize dignity, relational autonomy, and the need to balance technological intervention with compassionate presence.

From a digital health systems perspective, our findings indicate that technological maturity alone is insufficient to ensure transformation. Sustainable innovation in PC seems to require coordinated multilevel action, encompassing policy-level investment in infrastructure and funding stability, organizational leadership that legitimizes change and protects time for learning, interoperable digital systems that facilitate continuity across care settings, and professional development programs aligned with the relational values central to PC practice. Participants’ reflections also highlight the importance of co-design approaches involving frontline professionals and caregivers, ensuring that innovation initiatives remain grounded in everyday clinical realities. The findings also have practical implications for the design and implementation of innovation strategies in palliative care, highlighting the need for context-sensitive, human-centered approaches that align technological solutions with organizational capacity and professional practice. Importantly, innovation agendas should extend beyond digital tools to encompass psychosocial interventions, caregiver empowerment, and service reorganization, domains strongly emphasized by participants.

## 5 Conclusion

In conclusion, this exploratory study demonstrated that PC professionals across Europe are receptive to innovation, viewing it as a way to enhance communication, continuity, and family support. Despite this openness, most respondents perceived that implementation is often hindered by funding limitations, workforce pressures, and inconsistent institutional support. The gap between awareness and routine use of innovation mainly reflects systemic obstacles, not professional resistance. These findings may support the planning and contextual adaptation of future innovation initiatives in PC. Prospective implementation and sustainable progress require strong leadership, adequate resources, and context-sensitive approaches that prioritize person-centered care.

## Data Availability

The raw data supporting the conclusions of this article will be made available by the authors, without undue reservation.
