# Supplementary material for: Healthcare professionals’ perspectives toward innovation in palliative care across European settings: an exploratory mixed-methods study
Source: Front Public Health. 2026 Sep 23;14:1934362. doi: 10.3389/fpubh.2026.1934362 (PMC13642787; doi:10.3389/fpubh.2026.1934362)
Supplement: Supplementary file 1 [file Supplementary_file_1.DOCX]

Supplementary Material

# 1 Supplementary Data

## 1.1 Full Survey: Study on the Perception of Palliative Care Professionals Regarding Innovation, Barriers, and Facilitators

**Demographic and Professional Background**

1. **Age:**
   - 20-30
   - 31-40
   - 41-50
   - 51-60
   - 61+
2. **Gender:**
   - Male
   - Female
   - Other
   - Prefer not to say
3. **Country:**
4. **Regional Location:**
   - Castile and León
   - Aragon
   - Basque Country
   - New Aquitaine
   - Auvergne
   - Northern Portugal
   - Other (please specify)
5. **Occupation:**
   - Doctor
   - Nurse
   - Social Worker
   - Psychologist
   - Other (please specify)
6. **Years of Experience in Palliative Care:**
   - 0-5 years
   - 6-10 years
   - 11-15 years
   - 16-20 years
   - 21+ years
7. **Type of Institution:**
   - Hospital
   - Hospice
   - Home Care
   - Other (please specify)
8. **Institution Name:**

**Perceptions of Innovation**

9. **How would you rate the level of innovation in your palliative care setting?**
   - Very High
   - High
   - Moderate
   - Low
   - Very Low
10. **Which areas in palliative care do you think require the most innovation?** (Check all that apply)
    - Pain Management
    - Symptom Control
    - Communication with Patient
    - Family Support
    - Technological Integration
    - Training and Education
    - Other (please specify)
11. **What type of innovations do you believe could most improve patient care in palliative settings?** (Please describe)

**Barriers to Innovation**

12. **What do you perceive as the biggest barriers to implementing innovation in palliative care?** (Check all that apply)
    - Lack of Funding
    - Resistance to Change
    - Insufficient Training
    - Lack of Time
    - Regulatory Constraints
    - Other (please specify)
13. **To what extent do you agree with the following statement: "There is adequate support from management for innovation in my workplace."**
    - Strongly Agree
    - Agree
    - Neutral
    - Disagree
    - Strongly Disagree
14. **Have you faced any specific challenges when trying to introduce new practices or technologies in your work?** (Please describe)

**Facilitators of Innovation**

15. **What factors do you believe facilitate the adoption of innovation in palliative care?** (Check all that apply)
    - Strong Leadership
    - Collaboration and Teamwork
    - Funding Access
    - Supportive Policies
    - Patient and Family Engagement
    - Other (please specify)
16. **To what extent do you agree with the following statement: "Continuing education and professional development are encouraged and supported in my workplace."**
    - Strongly Agree
    - Agree
    - Neutral
    - Disagree
    - Strongly Disagree
17. **Can you share any examples of successful innovations that have been implemented in your palliative care setting?** (Please describe)

**Knowledge and Usage of Innovation Tools**

18. **Which of the following innovation tools are you aware of?** (Check all that apply)
    - Desktop or laptop computer
    - Email
    - Telephone/Fax
    - SMS text messaging
    - Mobile device (smartphone, tablet, wearable)
    - Social media (e.g., Facebook or Twitter)
    - Blog
    - Remote monitoring
    - Website browser
    - Wireless internet (Wi-Fi)
    - Wired internet
    - DVD player
    - Digital audio recording device
    - Electronic Health Records (EHR)
    - Telemedicine/Telehealth
    - Mobile Health Apps
    - Pain Management Devices
    - Artificial Intelligence (AI) for Predictive Analytics
    - Virtual Reality (VR) for Symptom Management
    - Robotics for Care Assistance
    - E-Learning Platforms
    - Other (please specify)
19. **Which of the following innovation tools do you use in your practice?** (Check all that apply)
    - Desktop or laptop computer
    - Email
    - Telephone/Fax
    - SMS text messaging
    - Mobile device (smartphone, tablet, wearable)
    - Social media (e.g., Facebook or Twitter)
    - Blog
    - Remote monitoring
    - Website browser
    - Wireless internet (Wi-Fi)
    - Wired internet
    - DVD player
    - Digital audio recording device
    - Electronic Health Records (EHR)
    - Telemedicine/Telehealth
    - Mobile Health Apps
    - Pain Management Devices
    - Artificial Intelligence (AI) for Predictive Analytics
    - Virtual Reality (VR) for Symptom Management
    - Robotics for Care Assistance
    - E-Learning Platforms
    - Other (please specify)
20. **How frequently do you use these innovation tools in your practice?**
    - Daily
    - Weekly
    - Monthly
    - Rarely
    - Never
21. **To what extent do you agree with the following statement: "The use of innovation tools has improved the quality of palliative care provided in my setting."**
    - Strongly Agree
    - Agree
    - Neutral
    - Disagree
    - Strongly Disagree
22. **Are there any innovation tools that you would like to use but currently do not have access to?** (Please describe)

**Purposes/Applications of Digital Health Technologies**

23. **For which of the following purposes do you use digital health technologies/applications?** (Check all that apply)
    - Communication with Patient
    - Symptoms Management
    - Medication Management
    - Medical Records
    - Teleconsultation
    - Continuing Training
    - Decision Support
    - Care Coordination
    - Other (please specify)
24. **How do you rate the effectiveness of digital health technologies/applications for the purposes mentioned above?**
    - Very Effective
    - Effective
    - Neutral
    - Ineffective
    - Very Ineffective
25. **Are there any additional purposes for which you would like to use digital health technologies/applications that are not currently available?** (Please describe)

**Openness to Innovation**

26. **How open are you to integrating new innovation tools into your palliative care practice?**
    - Very Open
    - Open
    - Neutral
    - Reluctant
    - Very Reluctant
27. **What factors would influence your willingness to adopt new innovation tools in your practice?** (Check all that apply)
    - Proven Effectiveness
    - User Friendliness
    - Costs
    - Training and Support Availability
    - Patient Preference
    - Collegues Recommendations
    - Institutional Support
    - Other (please specify)
28. **To what extent do you agree with the following statement: "I feel confident in my ability to learn and use new innovation tools."**
    - Strongly Agree
    - Agree
    - Neutral
    - Disagree
    - Strongly Disagree
29. **What support or resources would you need to feel more comfortable adopting new innovation tools?** (Please describe)

**Future Directions**

30. **In your opinion, what are the key steps needed to foster innovation in palliative care?**
31. **How do you see the future of palliative care evolving regarding innovation?**
32. **Do you have any additional comments or suggestions regarding innovation in palliative care?**

**Consent and Confidentiality**

- **Do you consent to participate in this survey and allow your responses to be used for research purposes?**
  - Yes
  - No

Thank you for your time and valuable input! Your responses will contribute to a better understanding of how to enhance palliative care through innovation.

# 2 Supplementary Table

## 2.1 STROBE (Strengthening the Reporting of OBservational studies in Epidemiology) checklist

**Supplementary Table 1.** STROBE Statement – checklist of items that should be included in reports of observational studies.

|  | **Item No.** | **Recommendation** | **Page  No.** | **Relevant text from manuscript** |
| --- | --- | --- | --- | --- |
| **Title and abstract** | 1 | (*a*) Indicate the study’s design with a commonly used term in the title or the abstract | 2 | “A cross-sectional mixed-methods survey study was conducted” |
|  |  | (*b*) Provide in the abstract an informative and balanced summary of what was done and what was found | 2 | “Survey data assessed (…) Findings suggest that” |
| **Introduction** | | | |  |
| Background/rationale | 2 | Explain the scientific background and rationale for the investigation being reported | 3 | “the integration of digital health technologies into PC presents unique challenges” |
| Objectives | 3 | State specific objectives, including any prespecified hypotheses | 4 | “we aimed to explore healthcare professionals’ attitudes toward digital innovation in PC across different European settings” |
| **Methods** | | | |  |
| Study design | 4 | Present key elements of study design early in the paper |  |  |
| Setting | 5 | Describe the setting, locations, and relevant dates, including periods of recruitment, exposure, follow-up, and data collection | 4 | “A cross-sectional mixed-methods study was conducted” |
| Participants | 6 | (*a*) *Cohort study*—Give the eligibility criteria, and the sources and methods of selection of participants. Describe methods of follow-up  *Case-control study*—Give the eligibility criteria, and the sources and methods of case ascertainment and control selection. Give the rationale for the choice of cases and controls  *Cross-sectional study*—Give the eligibility criteria, and the sources and methods of selection of participants | 4 | “health professionals working in PC” |
|  |  | (*b*) *Cohort study*—For matched studies, give matching criteria and number of exposed and unexposed  *Case-control study*—For matched studies, give matching criteria and the number of controls per case | N/A | N/A |
| Variables | 7 | Clearly define all outcomes, exposures, predictors, potential confounders, and effect modifiers. Give diagnostic criteria, if applicable | 4 | “(1) demographic and professional background; (2) perceptions of innovation; (3) barriers to innovation; (4) facilitators of innovation; (5) knowledge and usage of innovation tools; (6) purposes/applications of digital health technologies; (7) openness to innovation; (8) future directions” |
| Data sources/ measurement | 8 | For each variable of interest, give sources of data and details of methods of assessment (measurement). Describe comparability of assessment methods if there is more than one group | *4* | *“a set of questions was generated aiming to capture multiple dimensions of the theme”* |
| Bias | 9 | Describe any efforts to address potential sources of bias | 4 | “Category development was independently reviewed by two researchers, ensuring credibility and consistency in data interpretation.” |
| Study size | 10 | Explain how the study size was arrived at | 4 | “from January to July 2025” |

**Note:** An Explanation and Elaboration article discusses each checklist item and gives methodological background and published examples of transparent reporting. The STROBE checklist is best used in conjunction with this article (freely available on the Web sites of PLoS Medicine at http://www.plosmedicine.org/, Annals of Internal Medicine at http://www.annals.org/, and Epidemiology at http://www.epidem.com/). Information on the STROBE Initiative is available at www.strobe-statement.org
